# Preventive and protective measures reducing influenza transmission in general practice: a systematic review

**DOI:** 10.3399/bjgpopen19X101657

**Published:** 2019-08-21

**Authors:** Philipp Suter, Tessa Kermode, Carole Clair, Yolanda Mueller, Nicolas Senn

**Affiliations:** 1 Physician, Faculty of Medicine and Biology, University of Lausanne, Lausanne, Switzerland; 2 Physician, Unisanté — University Centre for Primary Care and Public Health, University of Lausanne, Lausanne, Switzerland; 3 Physician, Unisanté — University Centre for Primary Care and Public Health, University of Lausanne, Lausanne, Switzerland; 4 Physician and Lecturer, Unisanté — University Centre for Primary Care and Public Health, University of Lausanne, Lausanne, Switzerland; 5 Physician, Unisanté — University Centre for Primary Care and Public Health, University of Lausanne, Lausanne, Switzerland; 6 Professor, Unisanté — University Centre for Primary Care and Public Health, University of Lausanne, Lausanne, Switzerland

**Keywords:** Seasonal influenza infection, general practice, prevention, protection, infection control, transmission

## Abstract

**Background:**

Seasonal influenza and influenza-like illnesses are widespread, with an impact on GP consultations. GPs apply many preventive and protective measures to prevent seasonal influenza transmission, with no clear evidence of their effectiveness in this setting.

**Aim:**

To review the effectiveness of preventive and protective measures to reduce the transmission of seasonal influenza and influenza-like illnesses in GP practices.

**Design & setting:**

A systematic review was conducted of the literature in Medline, Embase, and the Cochrane Central Register databases published between January 1960 and April 2014, later extended to January 2018.

**Method:**

Preferred Reporting Items for Systematic Reviews and Meta-Analyses (PRISMA) criteria were used. Controlled trials and experimental studies were included. Study quality was assessed according to the Cochrane risk of bias tool.

**Results:**

Out of 5727 articles screened, only two studies were finally retained: one study about the seasonal influenza vaccination of GPs to prevent transmission from patients or staff, and one about surface disinfection. The first study was a controlled trial, which showed limited evidence for seasonal influenza infection reduction among GPs through vaccination. The second, an experimental study, performed a virus screening on toys in the waiting area before and after disinfection. No study on protection measures was found that assessed the impact on influenza transmission in general practices.

**Conclusion:**

The evidence is scarce on interventions that reduce influenza transmission in GP practices.

## How this fits in

There is currently insufficient evidence that staff vaccination or use of non-pharmaceutical protective interventions (NPIs) decrease seasonal influenza transmission in GP practices. More generally, there are limited data on the circulation and transmission patterns of influenza in GP practices. There is a need to better assess this, as well as to study preventive and protective measures specifically in GP practices, in order to make clear and adequate recommendations for this specific setting. In the meantime, it seems wise to recommend vaccination of staff and additional protective measures for healthcare workers (HCWs), by extrapolation from other healthcare settings.

## Introduction

Seasonal influenza is a potentially serious illness and is responsible for an important part of the burden of infectious diseases worldwide.^1,2^

The three (non-mutually exclusive) transmission routes for influenza are aerosols, droplets, and direct contact.^3–5^ All three transmission routes are present in the GP practice; for example, through close contact in the waiting area, desk surfaces, and the sharing of magazines with viral contamination.^3,5,6^ NPIs are of particular interest in cases of high-risk individuals responding poorly to vaccination or with contraindication to vaccination, or when vaccination coverage is unknown, such as in the waiting room.^7^ HCWs in practices often use NPIs as protective measures during the influenza season, including masks, disinfectant hand gel dispensers, disinfectant wipes, surface disinfection, or social distancing through isolation of ill patients.^8^

Influenza virus persistence on commonly-used equipment or desks is an important target for controlling viral transmission by contact.^3^ Many disinfection substances are proven for neutralising influenza virus on surfaces, but none has been specifically tested in practices.^9–11^ Furthermore, environmental factors, such as humidity and temperature, as well as individual factors, like aerosol production, may influence the transmission of influenza.^4^

HCWs are especially at risk for seasonal influenza infection.^12^ The relative risk ratio (RR) for unvaccinated HCWs to be infected is estimated to be three to four times higher than for comparable vaccinated adults.^13^ HCWs build up a high basic immunity and therefore more often have pauci- or asymptomatic seasonal influenza compared to their patients, making them more likely to transmit the disease.^3,13^ Some studies have demonstrated that influenza transmission can be reduced in hospitals, institutions, and in the community by preventive and protective measures, depending on the adherence rates to these measures.^12,14^ However, a Cochrane review showed no conclusive evidence for vaccinating HCWs to prevent seasonal influenza and its complications (lower respiratory tract infections [RTIs], hospitalisation, or death due to lower RTIs), or to reduce all-cause mortality in older people living in care institutions.^15^ Preventive measures are interventions before the illness appears and protective measures are interventions during the illness appearance. (Box 1)

Box 1 **Operational definitions^16^**

**Table:** 

Classification	Term	Definition
**Prevention**		Prevention has the goal of decreasing the impact of a predictable phenomenon. The prevention occurs before the problem appears.Prevention refers to measures and actions taken by an individual or a society to prevent disease happening or its consequences. In general, prevention includes a wide range of interventions aimed at reducing risks to health.
	**General**	General prevention, applied before the problem appears, reduces the risk of a specific group being affected by a phenomenon.
	**Personal**	Personal prevention, applied before the problem appears, reduces the personal risk of being affected, at the time of real exposure.
**Protection**		Protection has the goal of decreasing the impact of the phenomenon, but only comes into operation when the event is taking place. The protection is, in general, a physical intervention. The exposure to the event is reduced through the intervention.
	**General**	General protection provides protection to the whole population in the same room or environment in which the protection measure is placed.
	**Personal**	Personal protection provides a physical protection against an actual, real, existing phenomenon.

GPs are often the first medical contact for patients with RTIs and only a minority of patients need hospital care.^12^ Thus, GPs have a central and specific role to play in the prevention of influenza.^17^ Furthermore, HCWs in practices have many short periods of contact with patients with influenza-like illnesses during consultation or at the front desk, making this setting distinct from hospitals or the community.^13^

Most studies investigating the impact of preventive and protective measures are performed in hospital settings and results are extrapolated to other settings, such as general practices, without considering the potential difference in patterns of influenza transmission. Furthermore, most randomised-control trials (RCTs) are performed in ideal research conditions; the effectiveness of these interventions in day-to-day practice may therefore be questioned.

The present study aims to systematically review the literature on the evidence of the effectiveness of preventive and protective measures to reduce the transmission of seasonal influenza in practices.

## Method

A systematic literature review was performed of studies assessing preventative and protective measures targeting influenza transmission within practices. The MeSH terms (**Me**dical **S**ubject **H**eadings of the US National Library of Medicine) used are listed in Box 2. The inclusion and exclusion criteria are detailed in Box 3.

Box 2 **Research strategy: MeSH terms used**

**Table:** 

Influenza and prevention in primary care	
A Influenza	**MeSH terms:** ‘Human Influenza‘ [MeSH] OR ‘Influenza virus‘ [MeSH] OR ‘Influenza A Virus‘ [MeSH] OR ‘Influenza B Virus‘ [MeSH] OR ‘Influenza H1N1‘ [MeSH] OR ‘Influenza H2N3‘ [MeSH] **Natural wording:** flu
B Prevention	**MeSH terms:** ‘Prevention‘ [MeSH] OR ‘Vaccination‘ [MeSH] OR ‘Vaccine‘ [MeSH] OR ‘Immunisation‘ [MeSH] OR ‘Pharmaceutical Prophylaxis’ [MeSH] OR ‘Pharmaceutical Prevention‘ [MeSH] OR ‘Preventive Neuraminidase Inhibitor‘ [MeSH] OR ‘Complementary Medicine‘ [MeSH] OR ‘Hand Hygiene‘ [MeSH] OR ‘Hand Disinfection‘ [MeSH] OR ‘Isolation‘ [MeSH] OR ‘Administration Control‘ [MeSH] **Natural wording:** Neuraminidase inhibitor OR antiviral agents OR hand washing OR hand sanitizer OR patient isolation OR quarantine OR social distancing OR school closure OR inhalation of steam OR vapour inhalation OR humidified air OR sport OR exercise OR vitamin C OR vitamin D OR vitamin E OR tobacco cessation OR ginseng OR green tea OR echinacea purpurea OR meditation or hydrotherapy OR sea buckthorn berries OR gargling OR virucidal handkerchief
C Primary care	**MeSH terms:** ‘Primary Care‘ [MeSH] OR ‘General Medicine‘ [MeSH] OR ‘General Practitioner‘ [MeSH] OR ‘Family Medicine‘ [MeSH] OR ‘Ambulatory Care‘ [MeSH] OR ‘Community Medicine’ [MeSH] OR ‘Family Physician‘ [MeSH] **Natural wording:** PC OR family doctor OR PCP OR general practitioner office
**Influenza and protection in primary care**	
Influenza	**MeSH terms:** ‘Human Influenza’ [MeSH] OR ‘Influenza virus’ [MeSH] OR ‘Influenza A Virus’ [MeSH] OR ‘Influenza B Virus’ [MeSH] **Natural wording:** flu
Protection	**MeSH terms:** ‘Protection’ [MeSH] OR ‘Personal Protective Equipment’ [MeSH] OR ‘Protective Devices’ [MeSH] OR ‘Chirurgical Mask’ [MeSH] OR ‘Respirator’ [MeSH] OR ‘N95 Mask’ [MeSH] OR ‘Respiratory Protective Devices’ [MeSH] OR ‘Gloves’ [MeSH] OR ‘Surface Disinfection’ [MeSH] OR ‘Ventilation’ [MeSH] OR ‘Aeration’ [MeSH] OR ‘Air Filtering’ [MeSH] OR ‘Negative Pressure Room’ [MeSH] OR ‘Air Disinfection’ [MeSH] OR ‘Ultraviolet Irradiation’ [MeSH] **Natural wording:** PPE OR surface cleaning OR avoidance of handshaking OR blouse OR protective clothing OR ultraviolet radiation
Primary care	**MeSH terms:** ‘Primary Care’ [MeSH] OR ‘General Medicine’ [MeSH] OR ‘General Practitioner’ [MeSH] OR ’Family Medicine’ [MeSH] OR ‘Ambulatory Care’ [MeSH] OR ‘Community Medicine’ [MeSH] OR ‘Family Physician’ [MeSH] **Natural wording:** PC OR family doctor OR PCP OR general practitioner office

MeSH = **Me**dical **S**ubject **H**eadings of the US National Library of Medicine.

Box 3 **Inclusion and exclusion criteria**

**Table:** 

Inclusion criteria
**Type of study:** randomised controlled trials, quasi-experimental studies, observational studies (like cohorts, case-control, and cross-sectional studies), laboratory studies with clinical endpoint, before-and-after studies**Participants**: adults and/or older patients**Setting**: GP practice**Language**: English, French, or German**Abstract**: available**Outcome**: seasonal influenza infections, influenza-like illness with direct impact on transmission of influenza in GP practices, influenza virus transmission to HCW
**Exclusion criteria**
**Type of study:** case series, case reports, mathematical modelling, experimental laboratory studies or studies with no clinical endpoint (for instance, methods to get higher vaccination rates, such as reminders, postcards, etc), expert opinion, studies on the epidemiology of influenza infections (evolution of immunity, means of transmission, virus survival on various surfaces, etc). These studies kept for introduction and discussion.**Participants**: animals**Setting**: laboratory, intensive care unit**Language**: not any of English, French, or German**Abstract**: not available**Outcome**: non-influenza infections (such as, rhinovirus infections), pandemic influenza infections, influenza vaccination rates

If the same study was published several times, only the most recent version was included.

All studies performed in adult and older populations that considered protective and preventive measures with a direct impact on transmission of seasonal influenza and influenza-like illnesses in the general practice were included in this study. Initial search terms were broader than just ‘GP practice’, as the text might not clearly or explicitly mention the practice, resulting in the need for an adapted search strategy in order to be confident that all potential interventions were captured. The terms ‘home care’, ‘institutional’, ‘community’ and ‘hospital setting’ were used.

The authors searched Medline, Embase, and the Cochrane Central Register databases for studies, published between January 1960–April 2014, and where at least the abstract was published in English, French, or German. An update was performed with the same research criteria, screening studies published between May 2014–January 2018. A clinical trial registry (http://www.clinicaltrials.gov/) was searched to identify relevant unpublished trials. The reference lists of included studies were screened to identify supplementary relevant studies.

In the absence of validated definitions of prevention and protection, the authors developed ad hoc definitions based on the World Health Organization’s concepts.^16^ Box 1 provides the study’s operational definitions.

### Study selection and data extraction

Two independent reviewers (TK and PS) screened all titles and abstracts of potentially relevant articles for eligibility. The the full text of the article was read if the title and abstract review were appropriate according to the pre-defined inclusion criteria. Disagreements were resolved after consultation of two further reviewers (CC and NS). The study update was performed by the same researchers, using the same research method.

PRISMA guidelines from 2009 were used to perform a structured and high quality review.^18^

Two reviewers (PS and TK) independently assessed the level of evidence of the included studies using the Cochrane risk of bias tool for non-randomised studies of interventions (ROBINS-l) and the classification system published by the Centre for Evidence Based Medicine of the University of Oxford.^19–21^ Consensus group discussions involving all investigators resolved disagreements between the two reviewers.

The following information was extracted for all retained studies: population, setting, type of intervention, study design, control group, outcome, and results.^22^ Separately, the measurable effects were extracted, such as odds ratio or relative risk, if possible with the corresponding 95% confidence intervals (CI).

Finally, studies were retained describing interventions aimed at reducing the transmission of influenza specifically in the practice (GP practice being understood as the working place of GPs and potential coworkers such nurses, medical assistants, and administrative staff).

## Results

**Figure 1.**
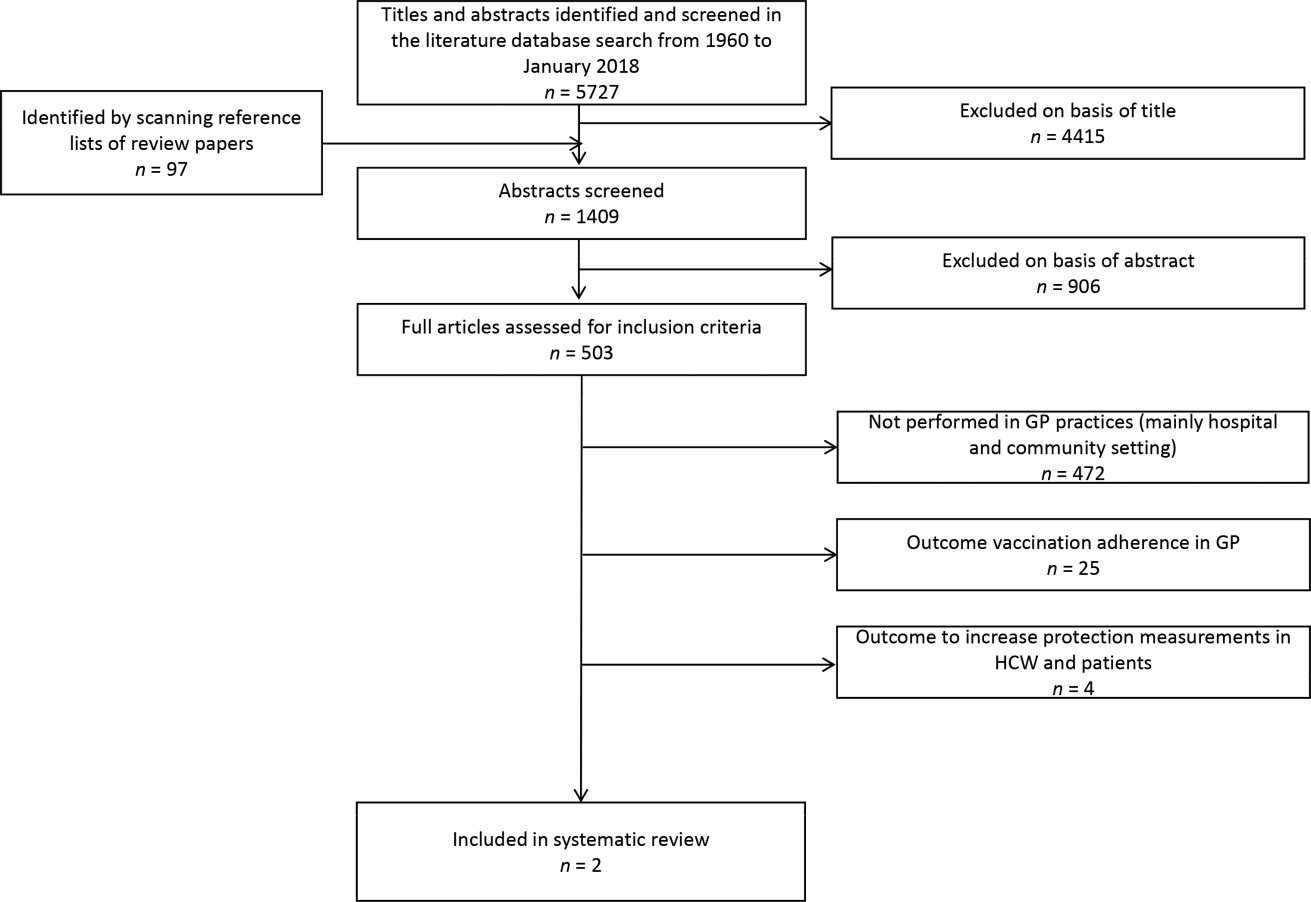
Flowchart of scoping

The research team identified 5727 citations from an initial screen of publications; 1409 were selected from the abstract screening, and an additional 97 studies or reviews were identified after screening the references of retrieved articles. Five-hundred and three were retained based on the abstract and full article assessment. Figure 1 is a flow diagram describing the selection process.

From the 503 retained studies, only two specifically addressed seasonal influenza transmission in practices: one RCT on vaccination effectiveness, by Michiels *et al*; and one observational study about surface disinfection, by Pappas *et al*.^12,23^ Out of the excluded 501 studies, 29 were performed in practice but with an outcome of vaccination adherence of GPs, or promotion of preventive or protective measures for HCWs or their patients in practice. The other 472 excluded studies, also assessing preventive or protective measures, were not performed in the general practice setting, but rather in hospitals, institutions, or in the community. No study combining prevention and protective measures was found. Table 1 presents the details of the two selected studies performed in GP practices.

**Table 1.** Summary of all data extraction results

Study	Type ofintervention	Control group	Population	Length offollow-up	ROBINS-I	Outcome	Effect
**Vaccination effectiveness**							
Michiels *et al* ^12^	Vaccination (TIV):*n* = 77 in 2002–2003;*n* = 100 in 2003–2004	No intervention:*n* = 45 in 2002–2003;*n* = 40 in 2003–2004	GPs working in Flanders	Influenza season 2002–2003; and 2003–2004	Moderate	SII by swab test, RTI, SII antibodies (4-fold hemagglutination inhibition antibody titre rise (taken once after the vaccination period but before the infection period; and taken once after the infection period)	**SII**: 8.6% in intervention vs 14.7% in control (RR 0.59; 95% CI = 0.28 to 1.24). **SII for ≤ 30-year-old GP** (OR 0.1; 95% CI = 0.01 to 0.75) **RTI**: 52.5% in intervention vs 53.3% in control (RR 0.98; 95% CI 0.76 to 1.27) **RTI for ≤ 30-year-old GP** (aOR 0.35; 95% CI = 0.13 to 0.96) **RTI and SII antibody rise**: 5.3% in intervention vs 18.8% in control (RR 0.28; 95% CI = 0.10 to 0.75) **RTI and SII and/or SII antibody rise**: 11.6% in intervention vs 26.1% in control (RR 0.44; 95% CI = 0.22 to 0.88) **RTI and SII and/or SII antibody rise for ≤30-year-old GP:** aOR 0.1; 95% CI = 0.02 to 0.98 **Low basic antibodies against SII predictive of SII**: aOR 0.57; 95% CI = 0.37 to 0.89 **Presence of influenza cases in the family predictive of SII:** aOR 9.24; 95% CI = 2.91 to 29
**Surface disinfection**							
Pappas *et al* ^23^	Samples on toys in ‘sick’ waiting room in GP practice before and after disinfection, samples on toys in new toy bag	Samples on toys in ‘well’ waiting rooms	5-provider (4 GPs and 1 nurse) general pediatric practice in northern Virginia	Respiratory virus season, 5 control dates	High	Picornavirus; influenza A and B; RSV	**Viral RNA detected on *n* = 11/52 (21%) of toys sampled:** *n* = 10 picoronavirus, *n* = 1 influenza B. **Viral RNA detected on *n* = 3/10 (30%) from the new toy bag**: *n* = 6/30 (20%) in ‘sick’ waiting room; *n* = 2/12 (17%) in ‘well’ waiting room. **Before cleaning the ‘ sick ’ waiting room:** *n* = 6/15 (40%) positive for picoronaviral RNA. **After cleaning:** *n* = 4/15 (27%); thus, RNA removed from *n* = 4/6 of the original positive. RNA was not transferred to the fingers of the investigator who handled theses toys.

aOR = adjusted odds ratio. CI = confidence intervals. OR = odds ratio. RNA = ribonucleic acid. RR = relative risk. RSV = respiratory synctial virus. RTI = respiratory tract infection. SII = seasonal influenza infection. TIV = trivalent influenza vaccination.

### Vaccination

No studies were found that directly tested the impact of vaccinating practice HCWs on influenza incidence among patients. Only one RCT measured the effectiveness of seasonal influenza prevention among GPs (in Flanders, during the two consecutive influenza seasons: 2002–2004). The quality of the study was evaluated as moderate, mainly due to missing data in the intervention group in the second wave. In this study, Michiels *et al* ^12^ compared 177 vaccinated GPs (intervention) with 85 non-vaccinated GPs (control). Outcomes were the occurrence of RTI and symptomatic swab-confirmed seasonal influenza during the influenza season, and four-fold increase in influenza haemagglutination inhibition antibody titre (comparing pre-infection period [post-vaccination titres in vaccinated] with after-infection period). There was no difference in the number of GPs with an RTI between the intervention and the control group (RR 0.98, 95% CI = 0.76 to 1.27), and a trend towards protection against seasonal influenza infection was observed in the intervention group compared to the control group (RR 0.59, 95% CI = 0.28 to 1.24). When comparing only the occurrence of RTI that included a four-fold influenza hemagglutination inhibition antibody titre, a 72% decrease in infection was observed between the intervention and control groups (RR 0.28, 95% CI = 0.10 to 0.75). In multivariate analyses, influenza vaccination of GPs aged ≤30 years was effective in preventing RTI, and seasonal influenza infection. GPs with a lower basic antibody titre against influenza and GPs with an influenza case in the family were more at risk for an episode of swab-positive seasonal influenza.^12^

### Protection

Only one experimental study was found about viral contamination in practice waiting areas; it did not study the effectiveness of the intervention on the seasonal influenza infection transmission rate.

#### Surface disinfection

In the patient waiting areas of one paediatric practice, one experimental study (before-and-after design) assessed contamination of toys by respiratory viral ribonucleic acid (RNA) during the respiratory virus season from October–March, before and after cleaning with disposable germicidal cloths. The quality of the study was evaluated as low. Twelve samples were taken in a ‘well’ waiting area and 30 samples in a ‘sick’ waiting area. The samples were taken in October, January, and March, but only the samples during the annual influenza epidemic in March were tested for influenza virus. Viral RNA was detected on 11/52 (21%) tested toys; only one was an influenza B virus. Fifteen toys were sampled before and after cleaning in October. Out of six samples positive for picornavirus, four became negative after cleaning, while two negative samples became positive. There is limited evidence, of low quality, that cleaning with disposable germicidal wipes eliminated viral RNA from toys.^23^

#### Combined non-pharmaceutical protection interventions (NPIs)

No studies were found assessing NPIs to specifically reduce the transmission of seasonal influenza in practices.

#### Pharmaceutical prophylaxis

No studies were found assessing prophylactic effect of pharmaceuticals on influenza transmission within practices.

## Discussion

### Summary

This systematic literature review investigated interventions aiming to decrease influenza transmission in practices. Only two studies were found that were specifically performed in practices. One study investigated the prevention of transmission of seasonal influenza through the vaccination of GPs, and the other was an interventional study about surface colonisation through respiratory viruses in waiting areas before and after surface disinfection. The study by Michiels *et al* showed a benefit of vaccinating GPs in practices, with a positive influence on seasonal influenza infection and RTI, although only among GPs aged ≤30 years.^12^ Pappas *et al*’s study about surface colonisation showed a high colonisation of toys by respiratory virus in the waiting area, but no impact of cleaning the toys. Overall, there is a lack of evidence to support any intervention aiming to decrease influenza transmission in practice.^23^

### Strengths and limitations

This systematic review was strengthened by a predefined search strategy, with a broad initial literature search followed by secondary filtering. Many relevant studies were captured by performing an additional reference list search.

The study has some limitations. First, definitions for prevention and protection had to be determined by the author group, as consensus definitions do not exist in the literature. This might limit the generalisability of the results. However, the authors tried to adhere as closely as possible to WHO recommendations, and believe that the operational definitions used might be useful and acceptable to a larger audience. Second, the wide literature search complicates the selection and interpretation of the studies, and the identification of those studies actually performed in general practice, and thus it is difficult to make clear statements about what might or might not work in general practices. On the other hand, a wide search has the advantage that all possible studied interventions were captured, independent of the setting, which thus provides a broad picture of what could be useful in practices. It also sheds light on the gaps in knowledge relating specifically to studies performed in this setting (that is, in general practices) and those extrapolated from other settings. A further limitation is that the research had to be updated a second time.

### Comparison with existing literature

Although vaccination of HCWs in practices may be an effective measure to reduce transmission of seasonal influenza between patients and HCWs, there are no studies directly assessing its effectiveness in this setting. The GP is in constant contact with patients and is, therefore, at special risk of transmitting seasonal influenza infection and RTI. Michiels *et al* showed that vaccinating GPs can protect them from contracting seasonal influenza.^12^ No study showed the influence of the GP or their patients’ vaccination status on seasonal influenza transmission in the practice. Several studies with high risk of bias were performed in nursing homes where HCWs were vaccinated against seasonal influenza. A Cochrane review did not find conclusive evidence in favor of vaccinating HCWs in care institutions.^15^ Indeed, investigating the effectiveness of vaccinating practice staff against seasonal influenza transmission is difficult, because symptoms are only expressed several days after infection, and it is difficult to conceive of a study design that would disentangle transmission in practice from that elsewhere in the community. Indeed, vaccinating patients against seasonal influenza in the practice, with trivalent or quadrivalent vaccination, may protect patients from seasonal influenza infection, but in this case transmission most likely occurs in the community.^24,25^

NPIs are implemented in the GP setting even though no study showed any real impact on RTI transmission in the practice. Pappas *et al* demonstrated the colonisation of toys in a waiting area with different respiratory infection viruses and a positive influence of cleaning the toys, but direct transmission to individuals handling the toys was not demonstrated.^23^ NPIs are mainly effective if used in combination.^26^ Again, as for vaccination, the effectiveness of most NPIs was mainly assessed in community settings and not in practices.^26^ For example, a significant risk reduction of RTI, influenza-like illness, or seasonal influenza was observed in studies that assessed seasonal influenza transmission in interventions combining mask use and hand hygiene in a community setting.^26–28^ A tendency towards reduction in seasonal influenza infection, and hospitalisation due to seasonal influenza infection, was observed with proper hand hygiene.^28^

In addition, interventions are only effective when they are used by a high number of HCWs and patients. Unfortunately, most studies confirm a low vaccination coverage against seasonal influenza infection (of 30.2%–81.3%) among GPs and HCWs, which could contribute to the transmission of seasonal influenza in the practice.^29–33^ Interventions such as vaccination promotion programmes, email reminders, or free vaccination programmes may improve vaccination coverage of general practice workers.^34,35^ The main barriers to vaccination identified in general practice HCWs were lack of awareness of vaccination recommendations and belief of low risk of contracting seasonal influenza.^31–33^ Patient seasonal influenza vaccination is higher if physicians are themselves vaccinated and when the patient consults more often, in addition to external factors such as media campaigns.^30,33,36,37^ However, there is currently no evidence directly linking vaccination coverage of the HCWs in general practice or of their patients with a decrease in seasonal influenza transmission in practice.

Similarly, combined NPIs are well investigated and implemented in hospital, institutional, and community settings, showing a tendency towards reduction of seasonal influenza infection and influenza-like illness transmission from HCWs to patients, but studies in general practices are missing.^27,28^ Promotion of NPI use positively influences the habits of HCWs, resulting in an increase in their use.^8,14,35^ Actual recommendations are based on extrapolation and it seems reasonable to recommend that GPs use NPIs, even though no studies were performed in GP practices.

Recommendations to reduce seasonal influenza transmission in practices are usually derived from other settings, such as hospitals or institutions, as in the case of the UK flu plan 2017/18, which recommends strict vaccination coverage for eligible patients and practice HCWs, as well as adapted respiratory and hand hygiene.^38^ Despite this, practices are different from hospitals and institutions in many aspects.^39^ Practices probably concentrate more people with influenza-like illness symptoms in close proximity, such as in the waiting area. For these reasons, extrapolating results from other settings to the practice might be quite hazardous. But, at present, it is the only means by which to make recommendations for the GP setting.

### Implications for research

Good quality studies about seasonal influenza transmission in general practices are lacking, both for prevention by vaccination and chemoprophylaxis, as well as possible combined effect with NPIs. In addition, most of the few existing studies use non-uniform definitions of influenza-like illness, non-uniform methods of detection of seasonal influenza infection, or inconsistent testing protocols, thereby impeding a proper comparison and interpretation of the studies. Good quality interventional studies are needed to provide evidence for the existing recommendations.
